# Evaluating the Impact of the Covariance of the Friction and Potential Gradient on Describing the Infiltration Process

**DOI:** 10.1007/s11242-018-1141-z

**Published:** 2018-09-08

**Authors:** Myron van Damme

**Affiliations:** Delft University of Technology, Delft, The Netherlands

**Keywords:** Darcy, Richards’ equation, Diffusion, Infiltration

## Abstract

During infiltration of water in soil, menisci form at the interface of water, grains, and air in the pores, inducing suction due to surface tension. Due to the random distribution of interconnected pores of different sizes, characteristic of porous media, differences in suction and friction inside pores give a diffusing infiltration front. The process of infiltration is often simulated by solving Richards’ equation in which the water flux is calculated with Darcy’s law. Underlying Darcy’s law is the assumption that the gradients in flow potential and the flow resistance due to viscous forces are independent from each other. This paper shows that these parameters are dependent and negatively correlated. A new method for calculating flows in unsaturated porous media has been developed to evaluate the impact of the covariance on infiltration predictions. The results show that the impact is significant and leads to a reduction in infiltration rate and mean friction experienced during infiltration. The method thereby provides a physical explanation for the subdiffusion observed during water infiltration in soil and is consequently expected to provide more insights into the processes of infiltration.

## Introduction

Soil is characterized by a random distribution in pore sizes. During water infiltration, water menisci form at the interface of water, soil grains, and air in the pores, inducing suction due to surface tension . Differences in suction and friction inside pores give a diffusing infiltration front whereby the infiltration rate is higher in the smaller pores than in the larger pores. The process of infiltration is often simulated by solving Richards’ equation (Richards 1931) which follows from substituting Darcy’s law in the mass balance equation.

Darcy (1856) discovered that for fully saturated porous media, the volume flux of water in soil is linearly dependent on the potential gradient over the porous media. Under fully saturated conditions, gradients in potential are deterministic and given by the imposed boundary conditions (Bonilla and Cushmann 2000) . The friction experienced by a laminar flow inside a pore thereby corresponds with the friction of a flow in an enclosed capillary tube. The friction is consequently inversely proportional to the pore radius *R* [m] squared (Pfitzner 1976; Sutera and Skalak 1993). The mean flow velocity $\tilde{w}$ inside a water-filled pore with radius *R* is related to the deterministic spatial gradient in flow potential head $\frac{d\phi }{\mathrm{d}\eta }$ according to

1 $$\begin{aligned} \tilde{w} = -\frac{ g R^2}{8 \nu }\frac{\mathrm{d} \phi }{\mathrm{d}\eta } \end{aligned}$$

Here, *g* [${\hbox {m}}/{\hbox {s}}^2$] is the gravitational constant, and $\nu$ [${\hbox {m}}/{\hbox {s}}^2$] is the kinematic viscosity of water. For low-velocity flows, the potential $\phi$ approximates the pressure head. From Eq. 1 follows that the expected value for the flow velocities inside a saturated porous medium $E[\tilde{\varvec{w}}]$, which is characterized by the random distribution in pore radii **R**, follows from

2 $$\begin{aligned} E[\tilde{\varvec{w}}] = -\frac{g}{8 n\nu }E[\mathbf{R}^2]\frac{\mathrm{d} {\phi }}{\mathrm{d} \eta } \end{aligned}$$

where *n* denotes the porosity of the soil and the term $\frac{g}{8 n\nu }E[\mathbf{R}^2]$ resembles the expected value for the friction experienced by the flow in the water-filled capillary tubes (Dekking et al. 2005). For saturated soil conditions, the potential gradients $\frac{\mathrm{d} {\phi }}{\mathrm{d} \eta }$ in the pores are deterministic and independent from each other (Bonilla and Cushmann 2000). A hydraulic conductivity parameter is now defined which is given by

3 $$\begin{aligned} K = \frac{1}{E[\pi \mathbf{R}^2]}\frac{g\pi }{8n\nu }E\left[ \mathbf{R}^4\right] \end{aligned}$$

Multiplying the expected value for the flow discharge $\frac{1}{E[\pi \mathbf{R}^2]}E[\pi \mathbf{R}^2 \tilde{\mathbf{w}}]$ with the porosity *n* and the expected value for the moisture content $E(\theta )$ gives the volume flux of water *Q* [m/s]. It should thereby be noted that for fully saturated soils, the expected value for the moisture content is constant. Darcy’s law is found when multiplying the hydraulic conductivity *K* [m/s] with the deterministic gradient in pressure head, which gives the specific discharge *Q* [m/s], or

4 $$\begin{aligned} Q = -K \frac{\mathrm{d} \phi }{\mathrm{d} \eta } \end{aligned}$$

Unlike saturated soils, unsaturated soils are characterized by the presence of water menisci at the interface of air, water, and soil particles. Over water menisci, the surface tension of water induces a drop in pressure head resulting in suction. Young (1805) discovered that the drop in pressure head $\Delta \phi$ [m] over a water meniscus is inversely proportional to the pore radius *R* and follows from

5 $$\begin{aligned} \Delta \phi = -\frac{2\gamma }{\rho g R} \end{aligned}$$

Here, $\gamma$ denotes the surface tension of water, and $\rho$ [kg / m$^3$] denotes the density of water. In soil, pore radii are randomly distributed. Consequently, the drop in pressure head at the boundary $\Delta \phi$ results in a stochastic Dirichlet boundary condition which gives a stochastic potential gradient field (Bonilla and Cushmann 2000). Over the years, different methods have been developed to account for the effects of water menisci on the infiltration process.

In 1911, Green and Ampt attempted to simulate the infiltration of water into unsaturated soil by representing soil as a series of independent capillary tubes between which no water exchange is possible. For a closed off capillary tube with a fixed diameter, the gradient in pressure head which is driving the flow is given by the difference in pressure head at either end of the tube $\phi |_{\eta _2}-\phi |_{\eta _1}$ divided by the infiltration length *L* (Green and Ampt 1911). For a flow down an enclosed capillary tube, the cross-sectional averaged flow velocity $\tilde{w}$ in a capillary tube is given by

6 $$\begin{aligned} \tilde{w} = -\frac{ g R^2}{8 \nu }\frac{\phi |_{\eta _2}-\phi |_{\eta _1}}{L} \end{aligned}$$

Because Green and Ampt (1911) considered all tubes to be independent from each other, the infiltration profile follows from summing the individual solutions for the flow velocities $\tilde{w}$ after multiplying them with the respective probability of occurrence. Equation 6 shows that the infiltration rate increases with an increase in pore radius *R*. A more rapid infiltration in the wider tubes than in the smaller tubes gives an increase in pressure gradients between tubes. For a porous medium like soil, this is unrealistic and conflicts observations. A criticism of this bundle-of-tubes description is therefore that it does not account for cross-flows between tubes (Bartley and Ruth 1999). Recently developed approaches therefore attempt to account for the exchange of water between pores (Dahle et al. 2005; Talbot and Ogden 2008), making the flow properties in pores dependent on the flow properties in other pores. These methods are, however, limited by the accuracy with which the exchange of water between pores is determined. In conclusion, bundle-of-tubes methods highlight two important aspects. One: An independent description of tubes gives an inaccurate description of the infiltration process, and two: Due to the dependence of the suction on the pore radius, the gradient in pressure head changes from being deterministic to stochastic.

Richards (1931) took a continuum-based approach to modelling flows in partially saturated porous media. Richards’ starting point was the mass balance equation which states that the change in moisture content inside a control volume equals the nett flux into that control volume, or

7 $$\begin{aligned} \frac{\partial {\theta }}{\partial t} = -\frac{\partial Q}{\partial \eta } \end{aligned}$$

Here, the first term refers to the change in overall moisture content, $E[{{\theta }}]$ with time *t*, and the term on the right-hand side denotes the nett inflow of moisture. Richards (1931) then stated that the volume flux *Q* [m/s] could be described by Darcy’s law (see Eq. 4) leading to Richards’ equation which is given by

8 $$\begin{aligned} \frac{\partial { \theta }}{\partial t} = -\frac{\partial }{\partial \eta }\left( K E\left[ \frac{\mathrm{d} {\phi }}{\mathrm{d} \eta }\right] \right) \end{aligned}$$

where ${\phi }$ refers to the distribution in pressure heads. Equation 8 is closed by means of soil–water characteristic curves (Fredlund and Rahardio 1993; Fredlund and Xing 1994) which empirically relate the expected value in pressure head ${\phi }$ to the moisture content $\theta$. By applying Darcy’s law, Richards (1931) inherently assumed that the expected values of the viscous effects and the gradient in pressure head are independent of each other and that the pressure head is deterministic. The bundle-of-tubes description of soil has, however, shown that in unsaturated porous media, the viscous contributions to the friction and the gradients in pressure head are both stochastic parameters and dependent on the distribution in pore radii. In this case, the expected value for the flow follows from the product of the expected value of the hydraulic conductivity and the pressure gradient plus the covariance of the hydraulic conductivity and the pressure gradient (Dekking et al. 2005) or

9 $$\begin{aligned} E\left[ \tilde{\mathbf{w}}\right] = E\left[ \frac{g}{8n\nu }\mathbf{R}^2\right] E\left[ -\frac{\mathrm{d} {\phi }}{\mathrm{d}\eta }\right] +\text {Cov}\left( \frac{g}{8n\nu }\mathbf{R}^2,-\frac{\mathrm{d} {\phi }}{\mathrm{d}\eta }\right) \end{aligned}$$

where $E\left[ \tilde{\mathbf{w}}\right]$ denotes the expected value for the velocity inside a pore. Since the friction in a pore and the gradient in pressure head are negatively correlated, the covariance is negative. Applying Eq. 2 to unsaturated porous media hence gives an overestimation of the mean velocity. Similarly, the water flux *Q* through a porous medium follows from

10 $$\begin{aligned} Q = \frac{1}{E[\pi \mathbf{R}^2]}\left\{ E\left[ \frac{\pi g}{8n\nu }{} \mathbf{R}^4\right] E\left[ -\frac{\mathrm{d} {\phi }}{\mathrm{d}\eta }\right] +\text {Cov}\left( \frac{\pi g}{8n\nu }\mathbf{R}^4,-\frac{\mathrm{d} {\phi }}{\mathrm{d}\eta }\right) \right\} \end{aligned}$$

The friction in a pore and the gradient in pressure head are negatively correlated. Darcy’s law therefore overestimates the volume flux inside an unsaturated porous medium. The error introduced by applying Darcy’s law thereby equals the covariance of the viscous effects and the potential gradients multiplied by the surface area.

Studies have been performed on the characteristics of Richards’ equation in describing wetting fronts (Witelski 1996; Gilding 1991). According to these studies, Richards’ equation describes a continuously expanding wet region whereby the speed of wetting is finite. Several discrepancies have been found between the solution and experimental data (Nielsen et al. 1962; Bell and Nur 1978; Roeloffs 1988). To improve the predictive capacity of Richards’ equation, closed-form relationships have been developed that relate the hydraulic conductivity to the moisture content (Gardner 1958; van Genuchten 1980; Fredlund and Xing 1994) leading to a nonlinear partial differential equation. More recent improvements made to Richards’ equation have a basis in the field of thermodynamics and aim to provide more insights into the physical processes of infiltrations like soil hysteresis (Hassanizadeh and Gray 1980; Niessner and Hassanizadeh 2008). Caputo (2000) noted that the permeability of a soil diminishes with time as if the porous medium has a memory and went on to produce diffusion models by including memory formalism. The memory effect is attributed to chemical reactions between the porous medium and the pore fluid and due to the transportation of particles through the channels (Caputo 2000; Sapora et al. 2017). Pachepsky et al. (2003) introduced a dependence of the diffusivity on time or distance to account for the effects of the memory by replacing the first-order time derivative by a fractional time derivative. Fractional diffusion equations are related to random walk approaches and have shown to be a synthetic and efficient tool to model memory effects and non-local interactions (Scalas et al. 2000; Evangelista et al. 2011). Mathematical models based on fractional calculus have led to improved predictions of the time-dependent decrease in diffusion observed during experiments. These models thereby aim to incorporate the past history of the infiltration process on the present state of the system (Freitas et al. 2017). Although a formal agreement with experiments is obtained, the physical proof that the effects of memory are the source of the a time-dependent decrease in diffusion is still lacking.

Although these models do give an explanation for the time-dependent decrease in diffusion, they all are still based on substituting Darcy’s law in the mass balance equation and inherently do not yet account for the contribution of the covariance. This paper analyses the impact of the assumption underlying Darcy’s law that the covariance of the gradient in pressure head and the friction approximates 0. To perform this analysis, a new modelling methodology has been developed. In the new method, one mass and two momentum balance equations are solved to describe a problem in one spatial dimension. One of the momentum balance equations describes the flow through a pore, and the other momentum balance equation describes the exchange of water between pores. The momentum balance equations are linked to each other via the pressure, for which a pressure relationship is solved. At the location of water menisci, the method accounts for the discontinuities in pressure field. The number of parameters needed to solve these equations has been kept equal to the number of parameters needed to solve Richards’ equation. Soil–water characteristic curves are thereby used to derive a discretized pore space distribution (Fredlund and Xing 1994; Nimmo 2013) that serves as input for the model. The model output consists of a discretized distribution in flow velocities. The max flux then follows from the discrete sums of the discharges $w_j A_j$ in the pores multiplied by their respective probability $p_j$ and the porosity *n* and divided by the expected value for the surface area inside a pore.

11 $$\begin{aligned} Q = n\frac{\sum _{j=1}^{j=\infty }\tilde{w}_j A_j p_j}{\sum _{j=1}^{j=\infty }A_j p_j} \end{aligned}$$

Consequently, the model is well suited for evaluating the impact of the expected values. Section 2 describes the development of the new method. The results are, respectively, presented and discussed in Sects. 3 and 4.

## Methodology

Porous media are here characterized as a random distribution of interconnected pores of different sizes. Solutions for flows in porous media are therefore given by the statistical ensemble of solutions for the flow in and between each of the pores. The ensemble of pore sizes has been discretized into *k* distinct sizes, each with a distinct radius *R* [m] and corresponding probability *p* such that $\sum _{i=1}^{i=k}p_i=1$. The shape of a pore is assumed to be cylindrical which is in line with the bundle-of-tubes-like description (Dahle et al. 2005; Talbot and Ogden 2008). The porous flow of a liquid with density $\rho$ [kg / m$^3$] is consequently well described by the mass and momentum balance equations in cylindrical coordinates. In this section, the methodology is illustrated for the case of vertical infiltration of water into soil whereby the $\eta$ coordinate direction has been replaced with Cartesian $\hat{z}$ direction.

### Constitutive Equations

A laminar flow down the centre axis of a porous pipe is considered to have a parabolic velocity distribution given by

12 $$\begin{aligned} w(r) = \frac{2}{R^2}\tilde{w}\left( R^2-r^2\right) \end{aligned}$$

where *w* [m / s] is the flow velocity as a function of $r \in [0, R]$, where *R* [m] denotes the radius of the tube. The flow is maximum for $r=0$. The fraction at the right-hand side scales the parabolic distribution in such a way that the cross-sectional averaged flow velocity in the pore equals $\tilde{w}$.

#### Mass Balance

**Fig. 1:**
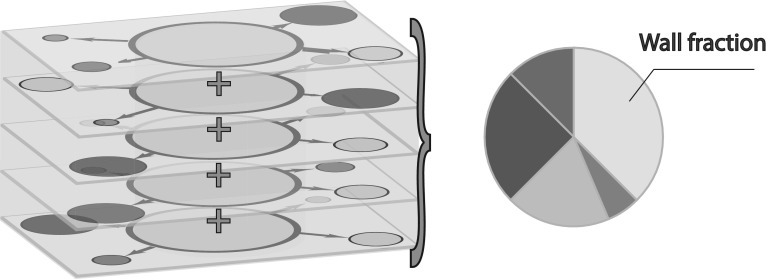
Representation of the wall fraction and neighbouring pore fractions of a porous medium divided into five discretized pore sizes (each pore has four neighbouring pores, or $j=4$)

The exact flow field inside a cylindrical-shaped tube is of little interest as the assumption of a cylindrical shape is only a simplification of the pore structure. This warrants integration over the cross section of a pore. Due to the porous nature of soil, each individual pore in a porous medium is connected with surrounding pores of different radii. The circumference of a pore, when represented as a cylinder, consists of a fraction of pore spaces denoted by the porosity *n* and a fraction of solid tube walls given by the grains and denoted by $(1-n)$. The pore fraction in turn consists of *j* fractions, each of which resembles a connection with another pore with a distinct pore diameter, such that the sum of the individual fractions leads up to 1. Consequently, $n p_i$ refers to that fraction of the circumference of the pore for which the pore is connected with a specific neighbouring pore denoted by index *i*. This has been illustrated in Fig. 1. Between pores, both mass and momentum are exchanged. The degree to which this exchange takes place depends on the probability of occurrence of the pores and their corresponding properties (See Fig. 1). Due to the presence of solid particles separating the pore spaces at the edge of the pore space, the flow in angular direction inside a pore is assumed negligible. The total exchange of water between pores is given by the discrete sum of the individual solutions for the flow between the pores. With *R* denoting the radius of the pore, $A = \pi R^2 = \sum _{i=1}^{i=k} \pi p_i R^2$, and with $\tilde{u}_{r}$ referring to the cross-sectional averaged nett outflow from the tube in radial direction, the cross-sectional integrated mass balance equation follows from integrating the mass balance equation in cylindrical coordinates over the cross-sectional surface area of a cylinder and is given by

13 $$\begin{aligned} \frac{\partial {\rho } A }{\partial t} +\frac{\partial \rho \tilde{w} A }{\partial z}+\sum _{i=1}^{i=k} 2\rho \pi R n p_i \tilde{u}_{r} = 0 \end{aligned}$$

Here, $p_i$ is the probability of occurrence of a surrounding pore, $\rho$ [kg / m$^3$] is the density of water and $\tilde{w}$ [m/s] is the cross-sectional averaged flow velocity down a pore.

#### Momentum Balance

For a laminar flow down a cylindrical pore, the shear stress is related to the flow velocity according to (Schlichting 1979)

14 $$\begin{aligned} \tau _{rz}=\mu \left( \frac{\partial w}{\partial r}\right) \end{aligned}$$

where $\mu$ $[{\hbox {Pa-s}}^{-1}]$ is the dynamic viscosity of water. Substituting Eq. 12 in Eq. 14 shows that the viscous effects experienced by the flow in a tube are linearly dependent on the cross-sectional averaged flow velocity in a tube $\tilde{w}$, which is in line with Darcy’s law, and Hagen–Poiseuille (Sutera and Skalak 1993). Consequently, integrating the momentum balance equation in cylindrical coordinates for flows in the $\hat{z}$ coordinate direction over the cross-sectional area of a cylindrical-shaped pore results in

15 $$\begin{aligned} \frac{\partial \rho A \tilde{w}}{\partial t}+\frac{\partial \rho A \tilde{w}\tilde{w}}{\partial z}+\sum _{i=1}^{i=k} 2 \rho \pi Rn \tilde{u}_r \tilde{w} p_i = -\rho A g_{z}-A\frac{\partial \tilde{P}}{\partial z}-8\pi \mu (1-n) \tilde{w} \end{aligned}$$

The friction experienced by the flow due to viscous forces is assumed to be due to the flow interaction with the solid particles, denoted by $(1-n)$ in the last term of Eq. 15.

The horizontal flow between tubes follows from integrating the radial momentum balance equation over the cross section of the tube. For the acceleration terms, this gives

16 $$\begin{aligned}&\frac{\partial \rho \tilde{u}_r A }{\partial t}+2\pi R \rho \tilde{u}_r \tilde{u}_r +\frac{\partial \rho \tilde{u}_r w A }{\partial z}\nonumber \\&\quad =\sum _{i=1}^{i=k}\left( ABp_i-\int \limits _{r_1}^{r_2}2\pi r_i p_i \frac{\partial P}{\partial r}\mathrm{d}r\right) -2\pi R\frac{\rho g n}{K_r}\tilde{u}_r \end{aligned}$$

Here, *B* denotes the body force in radial direction. The last term on the right-hand side denotes the friction experienced by the radial flow. It should be noted that due to the unknown size of the connections between the pores in radial direction, the term $K_r$ [m/s], which resembles the local conductivity in a pore, is at this stage yet unknown.

#### Pressure Relationship

Due to the exchange of water in and out of the pores and the effects of this exchange on the pressure gradient, the cross-sectional integrated pressure in a pore is a function of the pore diameter and *z*. An expression for the pressure follows from the Poisson equation which is found by taking the divergence of the generic momentum balance equations. Integrating the resulting expression over the angular direction from 0 to $2\pi p_i$ and over the radial direction *r* from 0 to *R* gives

17 $$\begin{aligned}&2\pi R\frac{\partial \rho \tilde{u}_r}{\partial t}|_{r=R}+2\pi \frac{\partial \rho \tilde{u}_r \tilde{u}_r R}{\partial r}+2\pi R\frac{\partial \rho \tilde{u}_r \tilde{w} }{\partial z} +\sum _{i=1}^{i=k}2\pi p_r RB+\frac{2\pi R\rho g n }{K_r}\tilde{u}_r\nonumber \\&\quad +\frac{\partial }{\partial z}\left( \frac{\partial \rho A \tilde{w} p_i}{\partial t}\right) +\left( \frac{\partial 2\pi p_i\rho \tilde{u}_r \tilde{w} R}{\partial z}\right) +\frac{\partial ^2 \rho A p_i \tilde{w}\tilde{w}}{\partial z^2}-A p_i\rho _w g\nonumber \\&\quad +\frac{\partial }{\partial z}\left( 8\pi \mu (1-n)\tilde{w}\right) = -\sum _{i=1}^{i=k}2\pi p_iR \frac{\partial \tilde{P}}{\partial r}|_{r_2}-A \frac{\partial ^2\tilde{P}}{\partial z^2} \end{aligned}$$

It has thereby been assumed that $\tilde{u}_r|_{r=0}=0$ and that *A* and *p* are constant in time.

### Integrating over the Infiltration Length

At this stage, each pore in a porous medium is assumed to be connected with few surrounding pores. Due to the random arrangement of the soil matrix and corresponding random distribution in pore sizes, for each pore it is unknown what the sizes of the directly adjacent pores are. Consequently, no local information is available on the pressure in each of the directly adjacent pores, or on the friction experienced by the flow towards or from the adjacent pores. This inhibits quantifying the flow exchange to and from each pore individually. Joekar-Niasar et al. (2008) addressed this problem by resolving to a network model in which the pore structure is randomized. Here, this problem is addressed by integrating each specific pore diameter over the infiltration length. Each individual pore size is thereby evaluated on the sum of the inflow and outflow from and to other pore sizes. It is thereby assumed that, over the length of infiltration, each specific pore size is connected with the full distribution of pore sizes larger and smaller than the pore size under evaluation. After removing the pore size under evaluation, the distribution of surrounding pore sizes is obtained. The cumulative sum of the probabilities of occurrences of the surrounding pore sizes per definition adds up to one, which has been accounted for by normalizing the probability of each surrounding pore $p_i$ by the discrete sum of the probabilities of the surrounding pores.

18 $$\begin{aligned} \tilde{p_i} = \frac{p_i}{\sum _{j=1}^{j=k}p_j|j\ne i} \end{aligned}$$

Each time, the flow properties that correspond to a single pore radius are evaluated. Pore radii are thereby not assumed to vary in time or space.

#### Mass Balance Integrated

Integrating the mass balance equation in each discrete pore size over the infiltration length in $\hat{z}$ direction from $z_1$ to $z_2$, where $z_1$ denotes the soil surface and $z_2$ the infiltration front, gives after applying Leibniz integration rule

19 $$\begin{aligned} \rho A\frac{\partial L}{\partial t}+\sum _{i=1}^{i=k}2\pi \rho R p_i \overline{\tilde{u}}_r L -A \tilde{w}|_{z_1}=0 \end{aligned}$$

By integrating each pore size over the infiltration length, the density no longer depends on *z*. The overbar $\overline{.}$ in Eq. 19 denotes the infiltration length averaged value. A new outflow parameter is now defined as $\tilde{q}_i = \overline{\tilde{u}}_r L$. Applying this parameter to the over the infiltration length integrated mass balance gives

20 $$\begin{aligned} A\frac{\partial L}{\partial t}+2\pi R \tilde{q}_r - A\tilde{w}|_{z_1}&=0 \end{aligned}$$

#### Momentum Balance Integrated

A positive consequence of integrating over the infiltration length is that the exchange of water between the pores occurs over the full distribution of pore sizes. The pressure gradient between two distinct pores averaged over the infiltration length is considered deterministic as the pores are fully saturated over the infiltration length. Hence, the volume flux between the pores could be determined using Darcy’s law whereby the average friction experienced by the flow is denoted by the saturated hydraulic conductivity $K_s$ [m/s]. Integrating the radial momentum balance equation over *z* from $z_1$ to $z_2$ now gives

21 $$\begin{aligned} \frac{\partial \rho \tilde{q}_r A }{\partial t}+2\pi R_i \rho \overline{\tilde{u}}_r \tilde{q}_r = \sum _{i=1}^{i=k} A B p_i L-\sum _{i=1}^{i=k}\int \limits _{r_1}^{r_2}2\pi R p_i L\frac{\partial P}{\partial r} \mathrm{d}r-\frac{A\rho g n }{K_s}\tilde{q}_r \end{aligned}$$

The integral of the pressure gradient in radial direction given in Eq. 21 yet needs to be determined. Applying integration by parts gives

22 $$\begin{aligned}&\sum _{i=1}^{i=k}\int \limits _{r_1}^{r_2}2\pi R p_i L \frac{\partial \overline{P}}{\partial r}\mathrm{d}r = \sum _{i=1}^{i=k}2\pi R p_i P L -\sum _{i=1}^{i=k} \int \limits _{r_1}^{r_2}P 2\pi \frac{\partial r L p_i}{\partial r}\mathrm{d}r \nonumber \\&\quad =\sum _{i=1}^{i=k}2\pi R p_i PL - \sum _{i=1}^{i=k}\int \limits _{r_1}^{r_2}\left( P 2\pi p_i L +P 2\pi R L \frac{\partial p_i }{\partial r}\right) \mathrm{d}r \nonumber \\&\quad = -E(2\pi RL\overline{\tilde{P}} )|_{r_2} \end{aligned}$$

Here, *E* denotes the expected value. Hence, when integrating the pressure gradients over the domain [0, *R*], the sum of the integral of the pressure gradients becomes equal to the expected value for the nett driving pressure multiplied by the area of each pore. When $\frac{\partial p_i}{\partial r}=0$, as would be the case for a uniform-distributed pore sizes, then no pressure differences are present between the pores and the flow in horizontal direction is 0.

#### Pressure Relationship Integrated

Equation 17 is now integrated over the $\hat{z}$ coordinate direction. Hereby, $\frac{\partial L}{\partial t} = w|_{z_2}$, and $\tilde{u}_r|_{z_1}=0$. Water is thereby assumed incompressible leading to

23 $$\begin{aligned} A w|_{z_2} -A w|_{z_1} +2\pi R \tilde{q}_r = 0 \end{aligned}$$

Spatial gradients in flow accelerations in groundwater flows are small allowing for gradients in advective acceleration to be omitted. Furthermore, substituting $Z=-\rho g$ and substituting Eq. 23 in Eq. 17 give an expression for the over the infiltration length averaged pressure inside a pore $\overline{\tilde{P}}$. This expression also guarantees that mass is overall conserved during the exchange of water between the pores.

24 $$\begin{aligned}&\sum _{i=1}^{i=k}2\pi p_i R L B|_{r_2}+\frac{\rho g n 2\pi R}{K_s}\tilde{q}_r|_{r_2} -\left( \frac{16\pi }{R} \mu (1-n)p_i+4\pi p_i \rho \overline{\tilde{u}}_r\right) \overline{\tilde{q}}_r \nonumber \\&\quad =-\sum _{i=1}^{i=k}2\pi p_i R\frac{\partial \overline{\tilde{P}}L}{\partial r} -A \left( \frac{\partial \tilde{P}}{\partial z}|_{z_2}-\frac{\partial \tilde{P}}{\partial z}|_{z_1}\right) \end{aligned}$$

For each pore size, the distribution of the surrounding pores is unique as the pore size under investigation is removed from the pore size distribution (see Fig. 1). This leads to *k* unique equations for *k* pore sizes, and it becomes possible to resolve the flow for each individual pore.

#### Body Forces

**Fig. 2:**
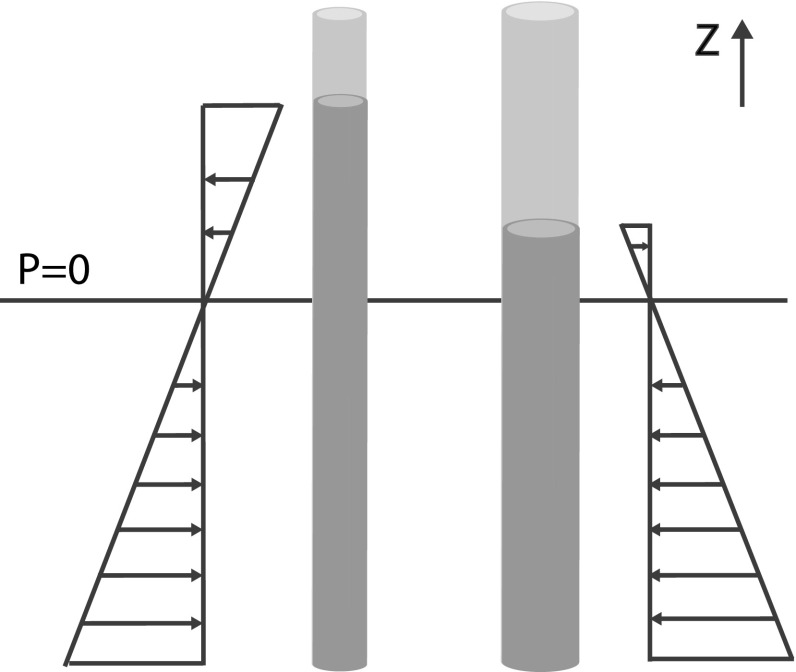
Pressure distributions for steady-state cases with capillary rise for two different pore radii

The final step needed to solve the mass and momentum balance equations is an expression for the body force *B*. For a situation at rest, the capillary rise is inversely proportional to the pore radius. Pressure profiles thereby linearly decrease over the capillary rise height and the mean pressure is $\overline{\tilde{P}} = \frac{\tilde{P}|_{z_1}+\tilde{P}|_{z_2}}{2}$ (see Fig. 2). With no radial body forces (*B*) in place, differences in boundary pressure $P|_{z_2}$ cause a net horizontal force which results in a flow between tubes of different diameters despite an equal pressure gradient in the pores, and an equal bottom boundary pressure (see Fig. 2). Consequently, a body force must be in place to correct this. A body force in horizontal direction could only originate from the capillary forces on that fraction of the sides of the pores in contact with air over the height equal to the difference in infiltration length between pore radii. Hence, the body force is a function of $P(L_i-L)$ where *L* is the pore under investigation and $L_i$ denotes the infiltration length in a pore with index *i*. Between two pore sizes, the pressure acting over the height $L-L_i$ would equal the average in pressure boundaries given by $\frac{1}{2}\left( P|_{z_2}+P_i|_{z_2}\right)$. Hence, the integrated body forces are given by

25 $$\begin{aligned} A\sum _{i=1}^{i=k}B p_i L = 2\pi R\sum _{i=i}^{i=k}\left[ \frac{\left( P_{z_2}|_{i}+P_{z_2}\right) }{2}\left( L_{i}-L\right) \cdot p_{i}\right] \end{aligned}$$

The gradient in body forces with respect to *R*, as given in the integrated pressure Poisson equation (see Eq. 24), is given by

26 $$\begin{aligned} \sum _{i=1}^{i=k}2\pi p_i B LR = 2\pi \sum _{i=i}^{i=k}\left[ \frac{\left( P_{z_2}|_{i}+P_{z_2}\right) }{2}\left( L_{i}-L\right) \cdot p_{i}\right] \end{aligned}$$

Substituting Eqs. 25 and 26 in, respectively, Eqs. 21 and 24 gives, respectively, the radial momentum balance equation and pressure relationship used for resolving the volume flux between pore sizes. The equations have been solved numerically according to the method prescribed in “Appendix A.”

## Results

In this section, the numerical solutions of the equations from Sect. 2.2 are given for two cases. For both cases, the pore size distribution is based on the Van Genuchten equation. When the steady-state potential is expressed in terms of the pore radius by means of $\phi = \frac{2\gamma }{R}$, then this equation becomes (Fredlund and Xing 1994)

27 $$\begin{aligned} P(\mathbf {R}<R)= \left[ \frac{1}{1+\left( -s\rho g \frac{2\gamma }{R}\right) ^w}\right] ^m \end{aligned}$$

**Fig. 3:**
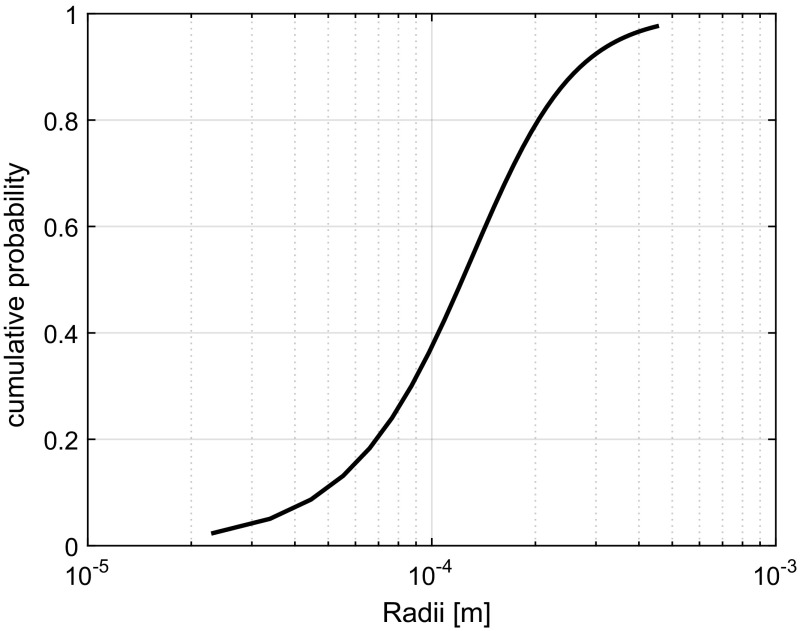
Pore space distribution corresponding to the demonstration case of horizontal infiltration

Here, *m*, *w*, and *s* are curve fitting parameters whereby $m =1-\frac{1}{w}$. The resulting distribution is depicted in Fig. 3 for $w=3$, and $s=1$. The values for $P(\mathbf {R}<R)$ range from $\frac{1}{\text {no}+2}$ to $\frac{\text {no}}{\text {no}+2}$, where $\text {no}$ is the discrete number of pore sizes indicative of the distribution. This results shown correspond with $\text {no} = 200$. A further increase in the number of tubes only provided a small increase in accuracy. The values corresponding with the lowest and highest cumulative probability have been used to, respectively, determine the upper and lower limit values for $\phi$, which have been converted into radii using Eq. 5. The radii are thereby assumed to increase at equal distant steps from $R_{\text {min}}$ to $R_{\text {max}}$.

**Fig. 4:**
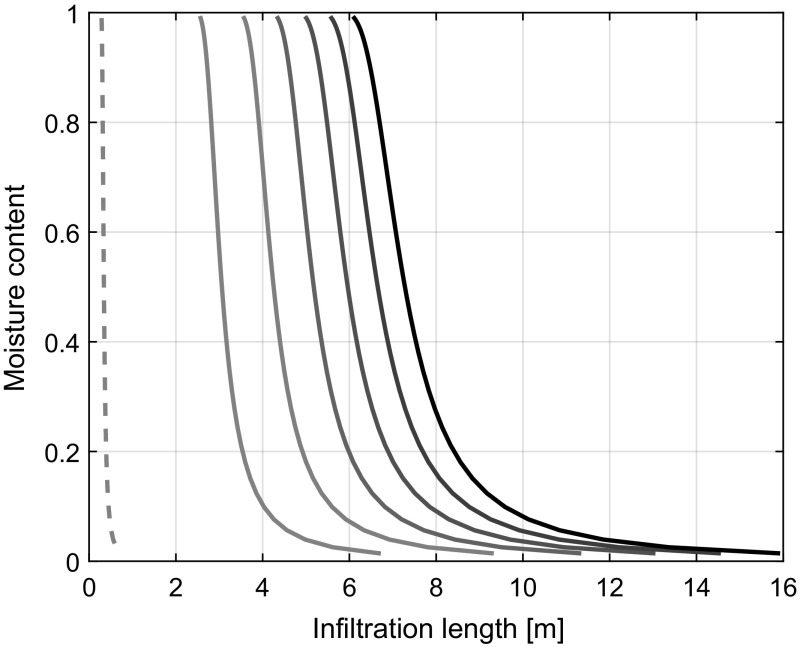
Moisture content profiles at 80 s intervals (from left to right) for the horizontal infiltration in soil. The dashed line indicates the initial infiltration profile

**Fig. 5:**
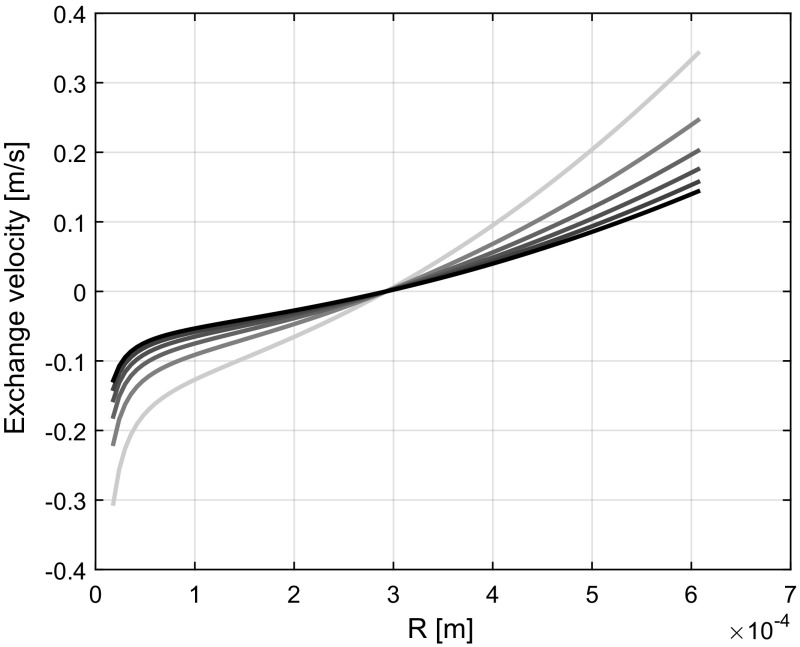
Volume exchange rate between pores divided by the cross-sectional area of a discretized pore taken at 80 s intervals (from light to dark) for the horizontal infiltration in soil. A positive velocity indicates an outflow from a pore. The initial condition consists of a 0 exchange

For various deterministic constant positive pressures described at a boundary location below a horizontal phreatic surface, the simulated capillary rise results in a rest saturation profile that corresponds with a moisture distribution given by Van Genuchten equation. The rate of capillary rise is thereby faster in the smaller pores than in the larger pores, indicating that the exchange of mass and momentum is accurately predicted by the model. Mass and momentum are thereby exchanged from the wider pores towards the narrower pores. Figure 4 demonstrates the model output for a case of horizontal infiltration in a soil with a pore space distribution given by Fig. 3. Here, the infiltration profiles are given at 80 s intervals whereby the outside water pressure is given by 0.50 m water column. For a higher positive pressure head, the degree of advection of the infiltration front was found to increase. The corresponding internal exchange of water between pores of different sizes is depicted for each of the time steps in Fig. 5. As can be seen, the larger pores show a net outflow and the smaller pores a net inflow. Water is hence rerouted from the larger pores towards the smaller pores. The exchange of water between the pores thereby decreases with time leading to the characteristic decrease in diffusion of the infiltration profile with time as illustrated in Fig. 4.

**Fig. 6:**
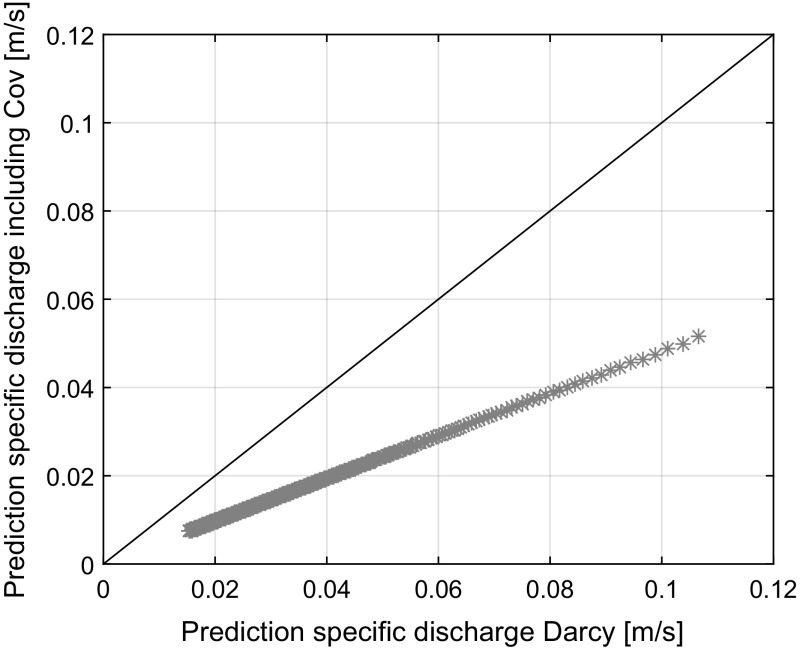
Product of the expected value of the potential gradient and the expected value of the friction (see Eq. 3) (horizontal) versus the expected value of the water flux (vertical). The difference indicates the error introduced by Darcy’s law in Richards’ equation

At the boundary where the soil is saturated, a water flux conforming to Darcy’s law is found by multiplying the expected value of the potential gradient by the expected value of the friction, both of which follow from the model. To determine the expected value for the friction which conforms to Darcy, the effects of the exchange of radial momentum were set at 0. The prediction of the specific discharge according to Darcy as depicted on the horizontal axis in Fig. 6 now represents

28 $$\begin{aligned} Q = \frac{1}{E[\pi \mathbf{R}^2]}\left\{ E\left[ \frac{\pi g}{8n\nu }{} \mathbf{R}^4\right] \right\} E\left[ -\frac{\mathrm{d} {\phi }}{\mathrm{d}\eta }\right] \end{aligned}$$

These results have been set out against the expected value for the mass flux that follows directly from the model, given on the vertical axis, which follows from

29 $$\begin{aligned} Q = \frac{1}{E[\pi \mathbf{R}^2]}\left\{ E\left[ -\frac{\pi g}{8n\nu }{} \mathbf{R}^4\frac{\mathrm{d} {\phi }}{\mathrm{d}\eta }\right] \right\} \end{aligned}$$

Figure 6 shows both predictions set out against each other for $t > 2$ s. During the first 2 s, the model is initializing. The initial conditions of the model were chosen such that no pressure gradients were present between the pores. After 2 s, the effects of this assumption were negligible. The difference in prediction indicates the effects of the covariance. The solid line denotes the perfect fit line. As time progresses, the nett effects of the covariance become smaller as indicated by the decrease in deviation from the perfect fit line when following the predictions in Fig. 6 from the top right to the bottom left. The relative effect, indicated by the ratio between predictions, however appears to remain constant.

**Fig. 7:**
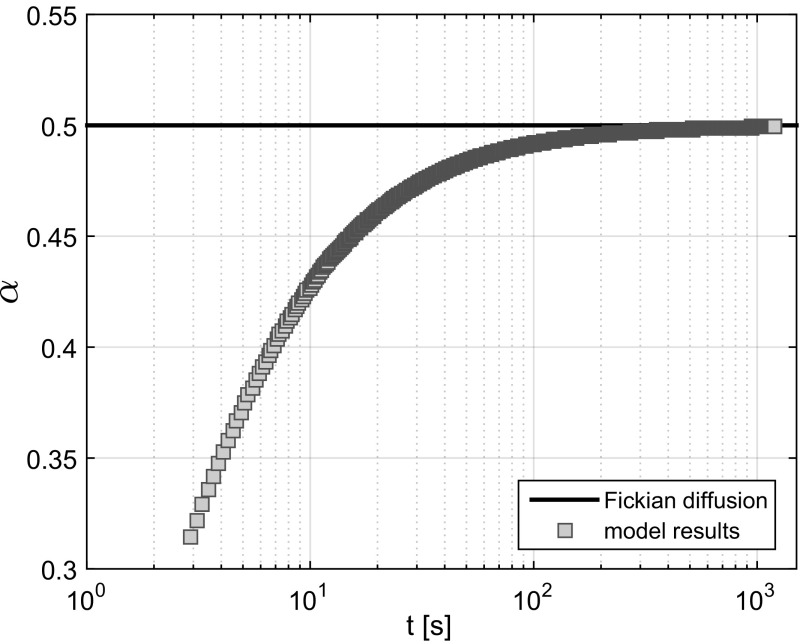
Change in subdiffusion with time

Substituting Darcy’s law in the mass balance equation gives Richards’ equations (see Eq. 8) (Richards 1931). By substituting the Boltzmann variable $\lambda = \frac{\eta }{t^{0.5}}$, Eq. 8 becomes

30 $$\begin{aligned} -\frac{\lambda }{2}\frac{\partial \theta }{\partial \lambda } = \frac{\partial }{\partial \lambda }\left( K(\theta )E\left[ \frac{\partial \theta }{\partial \lambda }\right] \right) \end{aligned}$$

where the hydraulic conductivity *K* is a function of the moisture content. For Eq. 30 to be valid, the relation between the expected value of the location of the infiltration front *L* and time *t* must obey $L::t^{\alpha }$ for $\alpha = 0.5$ (Pachepsky et al. 2003). Here, the value of $\alpha = 0.5$ denotes the Fickian diffusion of the infiltration front, indicating that the mean-squared displacement of the infiltration front is a linear function of time. Values for $\alpha <0.5$ indicate subdiffusion of the infiltration front whereby the mean-squared displacement of the infiltration front is a sublinear function of time. Defining $L_{\text {lg}} = {\text {lg}}(L)$ and $t_{\text {lg}}$ = ${\text {lg}}(t)$ gives that $\frac{\mathrm{d} L_{\text {lg}}}{\mathrm{d} t_{\text {lg}}} = \alpha$. Here, ${\text {lg}}$ refers to the natural logarithm. In Fig. 7, the values for $\alpha$ have been set out against time for $t > 2.8$ s. As indicated by the values for $\alpha < 0.5$, subdiffusion takes place during the initial stages of infiltration. Over time, the behaviour of the system converges to that of Fickian diffusion.

## Discussion

Pachepsky et al. (2003) noted that Richards’ equation is not general enough to simulate water transport in various soils. One of the improvements suggested by Pachepsky et al. (2003) was to introduce a dependence of the diffusivity on time or distance via a fractional derivative of the water content. This approach has been the focus of more recent studies which highlight the effectiveness of such an approach on capturing the subdiffusion of the infiltration front (Freitas et al. 2017; Evangelista et al. 2011; Sapora et al. 2017). Figure 7 shows that during the initial stages of infiltration, the change in expected location of the infiltration front with time is described by a subdiffusion process as $\alpha <0.5$, whereby $L::t^{\alpha }$. With time, the process asymptotically approaches a Fickian diffusion problem indicated by $\alpha = 0.5$. The values for $\alpha$ following from the model are in line with the values given by Pachepsky et al. (2003) who found values between $\alpha = 0.344$ and $\alpha = 0.479$ for infiltration depths up to 50 cm. Consequently, the model appears to capture the time-dependent diffusivity of the infiltration front well by simulating the exchange of mass and momentum between the pores. Richards’ equation follows from substituting Darcy’s law in the mass balance equation. An error is introduced in Richards’ equation by ignoring the impact of the covariance of the potential gradients and hydraulic conductivity in calculating the volume flux. Deviations from the Fickian diffusion indicated by the values for $\alpha <0.5$ are therefore indicative of the impact of the divergence of the covariance. The degree in deviation from Fickian diffusion during the initial infiltration (see Fig. 7) indicates that the effects of the divergence of the covariance are significant. It is important to note that the degree of subdiffusion decreases as the infiltration front progresses. Consequently, it is related to the degree of exchange of water between the different-sized pores and pressure differences between the pores. A more extensive study to quantify the effects of subdiffusion is recommended.

In the model, the water exchange between the different-sized pores follows from the mass and momentum balance equations. Pressure differences between the different pore sizes decrease as the infiltration front diffuses. This causes a reduction in the exchange of water from the larger towards the smaller pores and a decrease in subdiffusion of the infiltration front. Bundle-of-tube models (Talbot and Ogden 2008) and dual-permeability models (Gerke and Van Genuchten 1993) account for a redistribution of water between the different-sized pores. Characteristic of these types of models is that mass is redistributed, but momentum is not. In this type of models, the expected value of the pressure gradient in the individual pores is assumed to be independent on those in the surrounding pores. In the presented model, the pressures between the different pore sizes are made dependent on the other pores by solving the momentum balance equations for the flow between the pores. The presented model thereby shows similarities with the model of Sapora et al. (2017) who made the flow rate in a point dependent on the gradient in pressures in all nonadjacent points. Sapora et al. (2017), however, assumed that the sum of horizontal pressure differences is 0, indicating that the sum of the exchange of water between the pores is 0. Here is assumed that the sum of the exchange of water between the pores is equal to the sum of the spatial gradients in the exchange of water. Diffusion of the pressures between the different pore sizes and the time-dependent reduction in mass exchange between the different pore sizes thereby explain the subdiffusive behaviour of the infiltration front. The results may be used to extend the method of Sapora et al. (2017) by adding fractional time derivatives.

Figures 4 and 5 show the effect of the pressure differences between the different pore sizes and how diffusion of the pressure differences between the pores results in a continuous decrease in the rate of exchange of water between the different pore sizes. Diffusion of the rate of exchange implies that there is one pore size for which the nett inflow and outflow are the same. The rate of diffusion is thereby limited by the balance between the pressure differences and the flow resistance. A larger positive pressure head on the soil surface gives an increase in the rate of displacement of the infiltration front which corresponds with findings by Freyberg et al. (1980). During desorption, pressure differences between the pores change. Water exchange tends to be dominant in the direction of the larger pores towards the smaller pores due to the higher capillary action in the smaller pores. The lack of water exchange during desorption therefore results in a higher average flow resistance and a higher matric suction. This corresponds with the soil hysteresis phenomena described by Tami et al. (2004). To improve the understanding of the hysteresis phenomena, further studies to the dependence of pore pressure gradients and the exchange of water between the pores, indicated by the effects of the covariance, are therefore recommended.

A requirement for any model to capture the infiltration process correctly is the need to predict the volume flux correctly. Due to differences in pore sizes, the average infiltration rate is a function of the rate of exchange of water between the different pore sizes. In Richards’ equation, the average volume flux is determined by Darcy’s law which is based on the assumption that the friction in the pores and the potential gradient are independent on each other. The developed model was used to evaluate this assumption. Figure 6 shows that the impact of the dependence between the potential gradient and friction is significant. Ignoring the effect of the covariance gives an overestimation of the specific discharge predictions of approximately 48% (See Fig. 6). It should be noted that the quantification of this impact is influenced by the method of choice whereby pore sizes are averaged over the infiltration length and soil is schematized as a distribution of interconnected tubes. However, considering that the model physically captures the subdiffusion of the infiltration front indicates that in the case of unsaturated soils, the average pressures in a pore are dependent on those in the neighbouring pores. The effects of the dependence between the friction and capillary pressures on predicting the volume flux are thereby significant. The significant impact of the covariance also introduces questions about the impact of the continuum assumption underlying Richards’ equation (Gilding 1991). The problem with the continuum approach is that the divergence of the covariance cannot be accurately determined and the effects of the covariance on the inflow boundary conditions are a priori unknown. An advantage of the method presented herein over the Richards’ equation solvers is that the pressure boundary conditions and body forces are well defined in the model. The constitutive equations and solution method given here for the case of a 1D infiltration problem could thereby be extended to 2D or 3D problems. The model is thereby able to reproduce the subdiffusion of the infiltration front. Spatial and temporal fractional approaches may also be able to capture the impact more accurately. Further research on how to capture the effects of the covariance of the friction and potential gradient in these types of models is therefore recommended.

## Conclusion

This paper presents the results of a study to the impact of the dependence between the friction and potential gradient, which is ignored by Darcy’s law underlying Richards’ equation. Both the friction and potential gradient are a function of the pore radius and are randomly distributed. Darcy’s law must therefore be extended to include the covariance of the potential gradient and the friction. A new modelling method was presented which accounts for this dependence in simulating the infiltration process in unsaturated porous media. In this model, pore pressures have been made dependent on the pressures in the surrounding pores. Pressure differences between pores thereby balance the friction losses due to the exchange of water between the pores. The model output shows that ignoring the effects of the covariance gives a significant over-prediction of the volume flux and prevents the time-dependent diffusion process from being captured. The impact of the covariance, however, decreases as the infiltration depth increases. The degree of subdiffusion thereby approaches Fickian diffusion. Further studies to the impact of the divergence of the covariance are recommended to further improve the accuracy with which the infiltration process can be modelled.

## A Solution Method

This appendix describes the numerical scheme used to solve the mass and momentum balance equations given in Sect. 2.2. In the numerical solution, the spatial gradient in advective acceleration in the $\hat{z}$ direction has been ignored as this is small compared to changes in radial direction. The method of solving the equations has been based on an over the infiltration length integrated implicit pressure correction method (Chorin 1997; Ferziger and Peric 2002; Stelling and Zijlema 2003) which leads to the following steps.

1. Define the initial conditions
2. Use an initial guess for the averaged pressure $\overline{\tilde{P}}$ to solve Eq. 31. This results in a first guess for the over the infiltration length averaged radial flow velocity $\tilde{q}_r$[m/s].
  31 $$\begin{aligned}&\frac{\partial \rho \tilde{q}_r A }{\partial t}+\sum _{i=i}^{i=k}2\pi R p_i \rho \overline{\tilde{u}_r \tilde{q}_r} \nonumber \\&\quad = 2\pi R\sum _{i=i}^{i=k}\left[ \frac{\left( P_{z_2}|_{i}+P_{z_2}\right) }{2}\left( L_{i}-L\right) \cdot p_{i}\right] -E(2\pi RLP )|_{r_2}-\frac{\rho g n }{K_s}\tilde{q}_r \end{aligned}$$
3. Estimate the new infiltration length from
  32 $$\begin{aligned} A\frac{\partial L}{\partial t}+2\pi R \tilde{q}_r-A\tilde{w}|_{z_1}=0 \end{aligned}$$
4. Substitute the resulting value for $\tilde{q}_r$ in the pressure relationship given by Eq. 33 to arrive at a new estimation of a pressure field which also guarantees that mass is conserved.
  33 $$\begin{aligned}&2\pi \sum _{i=i}^{i=k}\left[ \frac{\left( P_{z_2}|_{i}+P_{z_2}\right) }{2}\left( L_{i}-L\right) \cdot p_{i}\right] +\frac{\rho g n 2\pi R}{K_s}\tilde{q}_r|_{r_2} -\frac{16\pi }{R} \mu (1-n) \tilde{q}_r \nonumber \\&\quad =-2\pi R \sum _{i=1}^{i=k}p_i \frac{\partial \overline{\tilde{P}}}{\partial r}-A \left( \frac{\partial \tilde{P}}{\partial z}|_{z_2}-\frac{\partial \tilde{P}}{\partial z}|_{z_1}\right) \end{aligned}$$
5. Repeat Steps 2 to 4 until the solution has converged
6. Solve the cross-sectional averaged momentum balance equation in the $\hat{z}$ coordinate direction to update the boundary condition for the inflow in each of the tubes.
  34 $$\begin{aligned} \frac{\partial \rho A \tilde{w}}{\partial t}|_{z_1}+8\pi \mu (1-n) \tilde{w}|_{z_1} = -\rho A g -A \frac{\partial \tilde{P}}{\partial z}|_{z_1} \end{aligned}$$
7. Go back to step one whereby the new initial conditions follow from the converged results of the previous time step.

### A.1 Discretization

Due to the very small distances between the pores, the momentum balance equations have been solved with the implicit time stepping method. The discharge $$\tilde{q}_r$$ and the pressure *P* are thereby solved iteratively. Below, the previous iteration step is denoted by the superscript $$^*$$. The result of the new iteration step is denoted by $$^{**}$$. The previous time step is denoted by superscript $$^{n}$$ and the new time step by superscript $$^{n+1}$$. The new time step is reached after the iterations have converged at which moment the variable at the new time step equals the last guess from the iterations. The length *L* has implicitly been accounted for in solving the radial momentum balance equation to improve model stability. The length at a new time step follows from the mass balance equation which has been discretized as

35 $$\begin{aligned} L^{n+1} =L^n-\Delta t \frac{2n}{R}\tilde{q}_r^{n+1} +\Delta t \tilde{w}^n|_{z_1}; \end{aligned}$$

please note that $\tilde{w}|_{z_1}$ [m/s] is defined at the old time step and is considered as a quasi-steady inflow boundary condition. The value for $\tilde{q}_r^{n+1}$ is given by the final iteration step for $\tilde{q}_r$. In the radial momentum balance equation (see Eq. 31), the local and advective acceleration term and the friction term have been discretized as

36 $$\begin{aligned} \rho A\frac{\tilde{q}^{**}-\tilde{q}^{n}}{\Delta t}+\left( 2\pi R p_i\frac{\tilde{q^*}}{L^*}+\frac{\rho g n}{K_s}\right) \tilde{q^{**}} \end{aligned}$$

A semi-implicit expected value for the driving force in the momentum balance equation in radial direction (See Eq. 31) follows from replacing the infiltration lengths by the expression for $L^{n+1}$ given by Eq. 35. This gives

37 $$\begin{aligned} -E\left( 2\pi R L \overline{\tilde{P}}\right) |_{r_2}&=-2\pi R \sum _{i=1}^{i=k}\left( \bar{\tilde{P}}_i^* L_i^{**}- \bar{\tilde{P}}^*L^{**}\right) p_i\nonumber \\&=2\pi R \sum _{i=1}^{i=k}\left( \bar{\tilde{P}}_i^* L_i^{**}p_i\right) - \bar{\tilde{P}}^{*}L^{**}\nonumber \\&=2\pi R \sum _{i=1}^{i=k}\left[ \bar{\tilde{P}}_i^* \left( L_i^{n}-\Delta t\frac{2n}{R}\tilde{q}_i^{**}+\Delta t \tilde{w}_i^n|_{z_1}\right) p_i\right] \nonumber \\&\quad -2\pi R \left[ \bar{\tilde{P}}^*\left( L^n-\Delta t\frac{2n}{R}\tilde{q}^{**}+\Delta t \tilde{w}^n|_{z_1}\right) \right] \end{aligned}$$

The body forces in Eq. 31 are represented by the capillary action at the side walls of the tube and are hence given by the difference in infiltration length between the tubes. The same procedure as followed for deriving the implicit discretized pressure relationship has been used to find an implicit discretized relationship for the body force. This results in

38 $$\begin{aligned} -&2\pi R\sum _{i=1}^{i=k}\left[ \left( \frac{P|_{z_2}+P_i|_{z_2}}{2}\right) \left( L^{**}-L_{i}^{**}\right) p_i\right] \nonumber \\&\quad =-2\pi R\sum _{i=1}^{i=k}\left[ \left( \frac{P|_{z_2}+P_i|_{z_2}}{2}\right) \left( L^{n}-L_{i}^{n}\right) p_i\right] \nonumber \\&\qquad -2\pi R\sum _{i=1}^{i=k}\left[ \left( \frac{P|_{z_2}+P_i|_{z_2}}{2}\right) \Delta t\left( \tilde{w}^n|_{z_1}-\frac{2n}{r}\tilde{q}_r^{**}-\tilde{w}_i^n|_{z_1}+\frac{2n}{R}\tilde{q}_{r}^{**}|_i\right) p_i\right] \end{aligned}$$

whereby $\sum _{i=1}^{i=k}p_i=1$. Hence, for tubes with a larger infiltration length than the tube under regard, the body forces are negative reducing the outflow from a tube, and for tube sizes smaller than the tube under regard, the body forces are positive giving a stronger outflow.

In the Poisson equation, the pressure terms consisting of the right-hand side of Eq. 24 have been discretized as

39 $$\begin{aligned}&-\sum _{i=1}^{i=k}2\pi p_i R\frac{\partial PL}{\partial r} - A \left( \frac{\partial P}{\partial z}|_{z_2}-\frac{\partial P}{\partial z}|_{z_1}\right) \nonumber \\&\quad =\sum _{i=1}^{i=k}2\pi p_i R \frac{\bar{\tilde{P}}_{i}^{**} L_i^{**}}{R}-2\pi p_i R \frac{\bar{\tilde{P}}^{**} L^{**}}{R} + 4\pi R^2\frac{\bar{\tilde{P}}^{**}}{L^{**}}-2\pi R^2\frac{P|_{z_2}}{L^{**}}-2\pi R^2\frac{P|_{z_1}}{L^{**}} \end{aligned}$$

Equation 39 has been solved by rewriting it as a matrix vector multiplication given by $$A \bar{\tilde{P}}^{**}=V$$. Matrix *A* thereby contains the known parameters and vector $$\bar{\tilde{P}}^{**}$$ the unknown pressures. Vector *V* follows from the input values given by the previous iteration step of the momentum balance equations and contains the pressure boundary conditions given by the last two terms in Eq. 39 and the parameters given by the left-hand side of Eq. 33 which have been discretized as

40 $$\begin{aligned}&2\pi \sum _{i=i}^{i=k}\left[ \frac{\left( P_{z_2}|_{i}+P_{z_2}\right) }{2}\left( L_{i}^{**}-L^{**}\right) \cdot p_{i}\right] +\frac{\rho g n 2\pi R}{K_s}\tilde{q}_r^{**}|_{r_2} -\frac{16\pi }{R} \mu (1-n) \tilde{q}_r^{**}; \end{aligned}$$

the total volume flux now follows from the sum of the flow down all tubes scaled by the area of the individual tubes.

41 $$\begin{aligned} q_z = \frac{1}{\sum _{i=1}^{i=k}A_i}\sum _{i=1}^{i=k}w_i p_i A_i n \end{aligned}$$

An advantage of the proposed method is that the exchange of water between the pores is based on the mass and momentum balance equations instead of an a priori assumed redistribution profile. The exchange of mass and momentum is thereby also accounted for when determining the overall redistribution of water in soil.
